# Supramolecular assembly of pentamidine and polymeric cyclodextrin bimetallic core–shell nanoarchitectures

**DOI:** 10.3762/bjnano.13.112

**Published:** 2022-11-18

**Authors:** Alexandru-Milentie Hada, Nina Burduja, Marco Abbate, Claudio Stagno, Guy Caljon, Louis Maes, Nicola Micale, Massimiliano Cordaro, Angela Scala, Antonino Mazzaglia, Anna Piperno

**Affiliations:** 1 Nanobiophotonics and Laser Microspectroscopy Center, Interdisciplinary Research Institute in Bio-Nano-Sciences, Babes-Bolyai University, T. Laurian Str. 42, 400271 Cluj-Napoca, Romania; 2 Department of Biomolecular Physics, Faculty of Physics, Babes-Bolyai University, M Kogalniceanu Str. 1, 400084 Cluj-Napoca, Romania; 3 Department of Chemical, Biological, Pharmaceutical and Environmental Sciences, University of Messina, V.le F. Stagno d'Alcontres 31, 98166 Messina, Italy; 4 National Council of Research, Institute for the Study of Nanostructured Materials (CNR-ISMN), URT of Messina c/o Department of Chemical, Biological, Pharmaceutical and Environmental Sciences, University of Messina, V.le F. Stagno d’Alcontres 31, 98166 Messina, Italy; 5 Laboratory of Microbiology, Parasitology and Hygiene (LMPH), University of Antwerp, S7, Universiteitsplein 1, 2610 Wilrijk, Antwerp, Belgium; 6 CNR-ITAE, Istituto di Tecnologie Avanzate per l’Energia, 98126, Messina, Italy

**Keywords:** antimicrobial agents, bimetallic nanoparticles, gold/silver core–shell, *Leishmania*, pentamidine, polycyclodextrin

## Abstract

Advanced nanoscale antimicrobials, originated from the combination of noble metal nanoparticles (NPs) with conventional antimicrobial drugs, are considered the next generation of antimicrobial agents. Therefore, there is an increasing demand for rapid, eco-friendly, and relatively inexpensive synthetic approaches for the preparation of nontoxic metallic nanostructures endowed with unique physicochemical properties. Recently, we have proposed a straightforward synthetic strategy that exploits the properties of polymeric β-cyclodextrin (PolyCD) to act as both the reducing and stabilizing agent to produce monodispersed and stable gold-based NPs either as monometallic (nanoG) structures or core–shell bimetallic (nanoGS) architectures with an external silver layer. Here, we describe the preparation of a supramolecular assembly between nanoGS and pentamidine, an antileishmanial drug endowed with a wide range of therapeutic properties (i.e., antimicrobial, anti-inflammatory, and anticancer). The physicochemical characterization of the supramolecular assembly (nanoGSP) in terms of size and colloidal stability was investigated by complementary spectroscopic techniques, such as UV–vis, ζ-potential, and dynamic light scattering (DLS). Furthermore, the role of PolyCD during the reduction/stabilization of metal NPs was investigated for the first time by NMR spectroscopy.

## Introduction

Noble metal nanoparticles (NPs), in particular those composed of gold and/or silver, are versatile agents endowed with unique physicochemical properties, which recently have drawn great interest for a variety of applications ranging from catalysis to nanomedicine [1–3].

The size of the NPs is a key parameter that defines their optical properties classifying them in plasmonic NPs (size > 5 nm) and nanoclusters (size < 5 nm). When dimensions exceed 5 nm, NPs exhibit a unique optical phenomenon called localized surface plasmon resonance (LSPR) which represents the collective oscillation of conduction band electrons after interaction between NPs and an electromagnetic field [4]. However, for <5 nm sized NPs, the LSPR phenomenon disappears, and they exhibit a tunable intrinsic photoluminescence with high Stokes shift and excellent photostability [5]. Plasmonic NPs can be produced in monometallic (MNPs) or bimetallic (BMNPs) forms and, in the latter, the internal distribution of the metallic element determines the structural characteristics of the resulting BMNPs (i.e., alloy, core–shell, or intermediate stages) [6].

The scientific value of noble MNPs and BMNPs has been recently highlighted by the development of novel antimicrobial agents with remarkable activity against bacteria, protozoa, and viruses [7–10]. They have been proposed to complement traditional antimicrobials, either to increase their potency or broaden their activity spectrum. Furthermore, their combination with classical antibiotics is considered a promising strategy to combat the ongoing public health threat of antimicrobial resistance [11].

Au/Ag BMNPs with different structural motifs and biological properties have been produced via eco-friendly synthesis using natural extracts or natural compounds which act as both reducing and capping agents [2]. When these green methods are used to obtain metallic NPs, particle growth, colloidal stability, as well as the biological profile of the resulting products are generally attributed to the phenolic and/or the carbohydrate components of the capping natural source [12].

Particularly interesting are Au/Ag bimetallic systems with a core–shell structure. In this case, the inner Au component favors cellular entry (typically via endocytosis) and slow release of the Ag^+^ ions, to which the overall biological activity of the system is ascribed. It all comes down to greater system biocompatibility [2].

Au/Ag BMNPs with a core–shell architecture can be obtained by the “seeded growth method” which consists in the sequential reduction of gold and silver ions. The co-reduction of the two metal ions (i.e., Ag^+^ and Au^3+^) produces an alloy instead [13].

**Figure 1:**
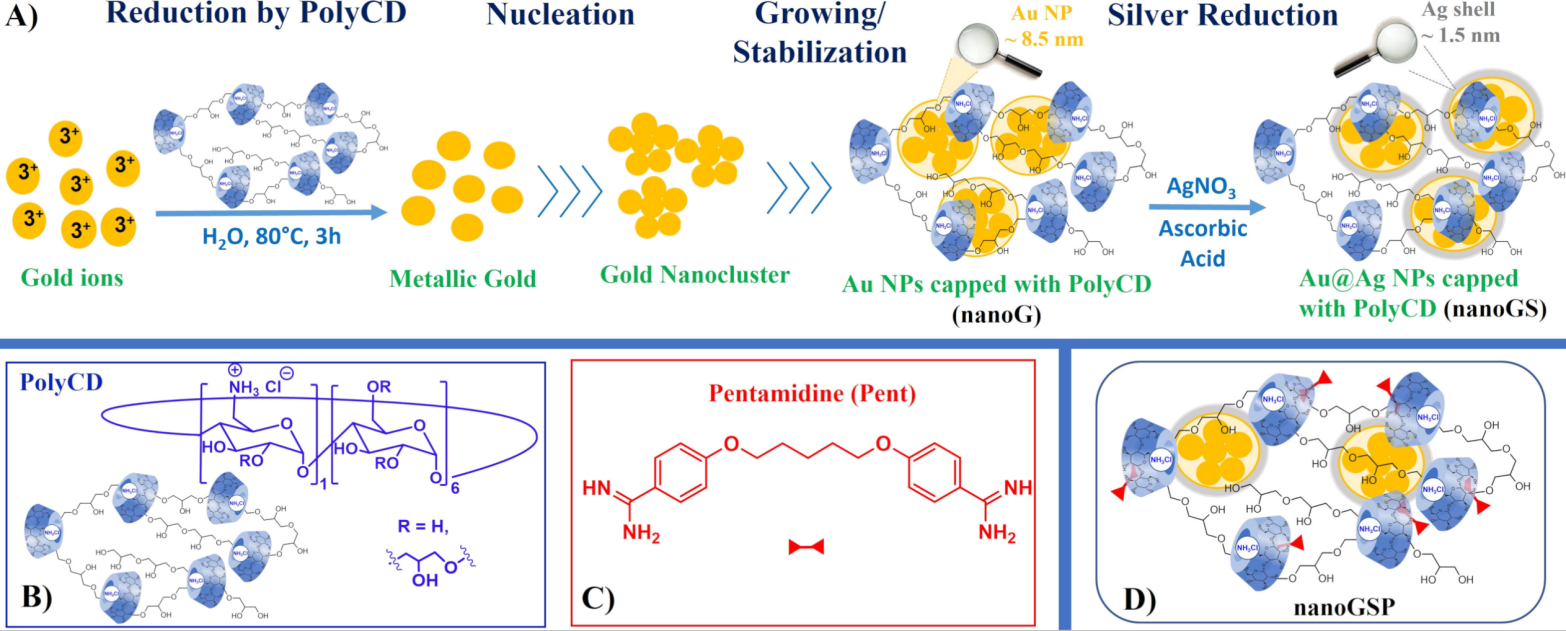
A) Schematic representation of the sequential reduction of gold and silver ions by PolyCD; B) chemical structure of PolyCD; C) chemical structure of Pentamidine; D) sketched view of supramolecular assembly between pentamidine and Au@Ag/PolyCD (nanoGSP).

In our ongoing research program aimed to the discovery of new antimicrobials, we recently explored the ability of the β-cyclodextrin polymer (PolyCD) to act as a symbiont of both MNPs and BMNPs [14]. Specifically, functional groups of PolyCD (i.e., amines, primary and secondary alcohols, and hemiacetals) are involved in the direct reduction of gold ions to Au NPs (Figure 1). PolyCD-capped Au NPs showed an impressive stability over time and over sterilization procedures, probably due to the colocalization of reduction and nucleation/growing sites during their formation. The stability was maintained or was found to be even higher in PolyCD-capped Au@Ag BMNPs, whereas monometallic Ag NPs produced from PolyCD in the same experimental conditions showed poor stability [14]. Although we are currently unable to elucidate the comprehensive mechanism that occurs during the reduction processes, we suppose that the direct reduction of gold ions by PolyCD takes place at sites involved in the nucleation/growing process of NPs and that this step is preparatory for the subsequent deposition of the silver shell. Conversely, in the case of monometallic Ag NPs, the reduction of silver ions (usually mediated by ascorbic acid) takes place at sites which are not involved in the nucleation/growing process leading to poorly stable Ag NPs. In the framework of our research on BMNPs, we collected several experimental data, including FTIR spectra, which suggested strong interactions between PolyCD and Au@Ag BMNPs [14]. In this paper we partially clarify the role of PolyCD in the formation/stabilization of Au@Ag BMNPs by distortionless enhancement by polarization transfer (DEPT)-edited heteronuclear single quantum correlation (HSQC) analyses. Moreover, taking advantage of the high ability of both CD cavities and silver layer to interact with small molecules to form nanoantibiotics [15], we decided to combine Au@Ag BMNPs (nanoGS) with pentamidine (Pent), an antimicrobial agent used against leishmaniasis [16], to investigate the antimicrobial activity of novel nanosystems (i.e., nanoGSP; Figure 1).

Leishmaniasis is an infectious disease caused by protozoan parasites transmitted to humans and animals by the bite of tiny (2–3 mm long) infected female insect vectors of the *Phlebotomine* subfamily (sandflies). It is an endemic disease in tropical and subtropical regions as well as in Southern Europe. According to the current WHO data, 50.000–90.000 new cases of visceral leishmaniasis [17] (the most severe form of this disease associated with high mortality rate if not promptly diagnosed and treated) are estimated to annually occur worldwide, predominantly in least developing countries due to poor hygiene, lack of protective measures (vector control), and lack of appropriate health infrastructures [18–19]. In addition, antileishmanial drugs which are currently available on the market showed several drawbacks including irreversible toxic effects, high costs, prolonged treatment, need for adequate medical care, emergence of drug resistance, and parenteral administration [20–21]. Recently, nanoantimicrobials based on biogenic noble metal NPs produced by reduction of gold or silver ions with natural extracts have been proposed for the treatment of leishmaniasis, acting against both promastigote and amastigote forms of *L. donovani* [22–23]. Nanoantimicrobials originated from the combination of NPs with conventional antimicrobial drugs are considered the next-generation antimicrobials, although their development is still confined to the lab bench due to NP colloidal instability [11,24].

In line with this research topic, herein we describe the preparation of Au@Ag NPs capped with PolyCD and loaded with Pent (i.e., nanoGSP, Figure 1) achieved by supramolecular assembly of the components as well as their physicochemical characterization in terms of size and colloidal stability. The drug binding ability of nanoGS with Pent has been investigated by complementary spectroscopic techniques such as UV–vis, zeta potential (ζ-potential), and dynamic light scattering (DLS). Experimental data suggested a multiple set of interactions between Pent and nanoGS that involves mainly the CD cavities. The biological profile of nanoG, nanoGS, and nanoGSP has been evaluated in terms of antileishmanial activity and cytotoxicity by in vitro assays against *L. infantum* and on MRC-5 cells and PMM cells, respectively, using a Pent-free base as the control.

## Results and Discussion

### Supramolecular assembly between pentamidine and polymeric cyclodextrin noble bimetallic core–shells

**Figure 2:**
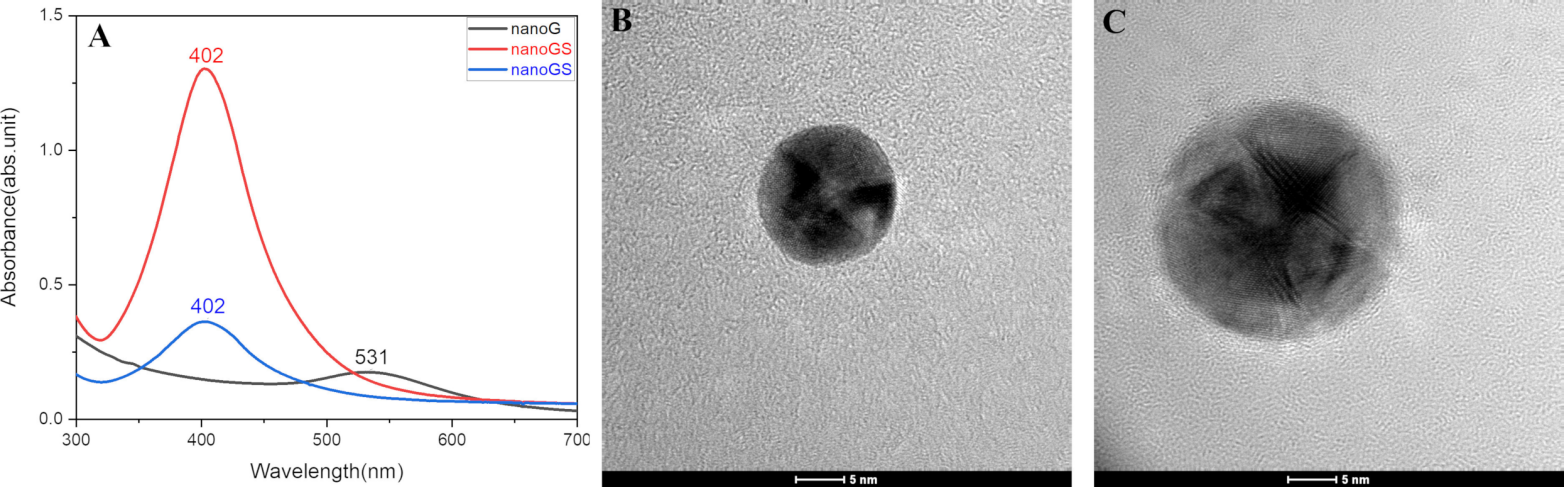
(A) UV–vis spectra of nanoG and nanoGS (red line: freshly prepared nanoGS; blue line: 4 times diluted nanoGS; *d* = 1 cm for nanoG and *d* = 0.2 cm for nanoGS). (B,C) TEM images of nanoG and nanoGS, respectively. Scale bar is 5 nm.

Gold/silver bimetallic NPs with core–shell architecture (nanoGS) were synthesized under mild conditions, according to our previously reported procedures [14], as follows: a) preparation of the gold inner core (nanoG) by direct reduction of HAuCl_4_ using PolyCD as both reducing and capping agent and b) deposition of an outer silver layer by ascorbic acid-mediated reduction of AgNO_3_. The formation of small gold NPs (Figure 2) was confirmed by the presence of the LSPR band detected at 531 nm in the UV–vis spectrum. The subsequent addition of AgNO_3_ resulted in a change of the extinction spectrum with the formation of a higher and broader absorption band at 402 nm (Figure 2). The morphology of nanoG and nanoGS in term of size, shape, and core–shell configuration was confirmed by TEM analyses. The gold NPs were spherical with an average size of 8 ± 3 nm, whereas the mean diameter increased to 11 ± 3 nm for core–shell NPs, resulting in the formation of a 1.5 nm silver shell (Figure 2B, Figure 2C).

**Figure 3:**
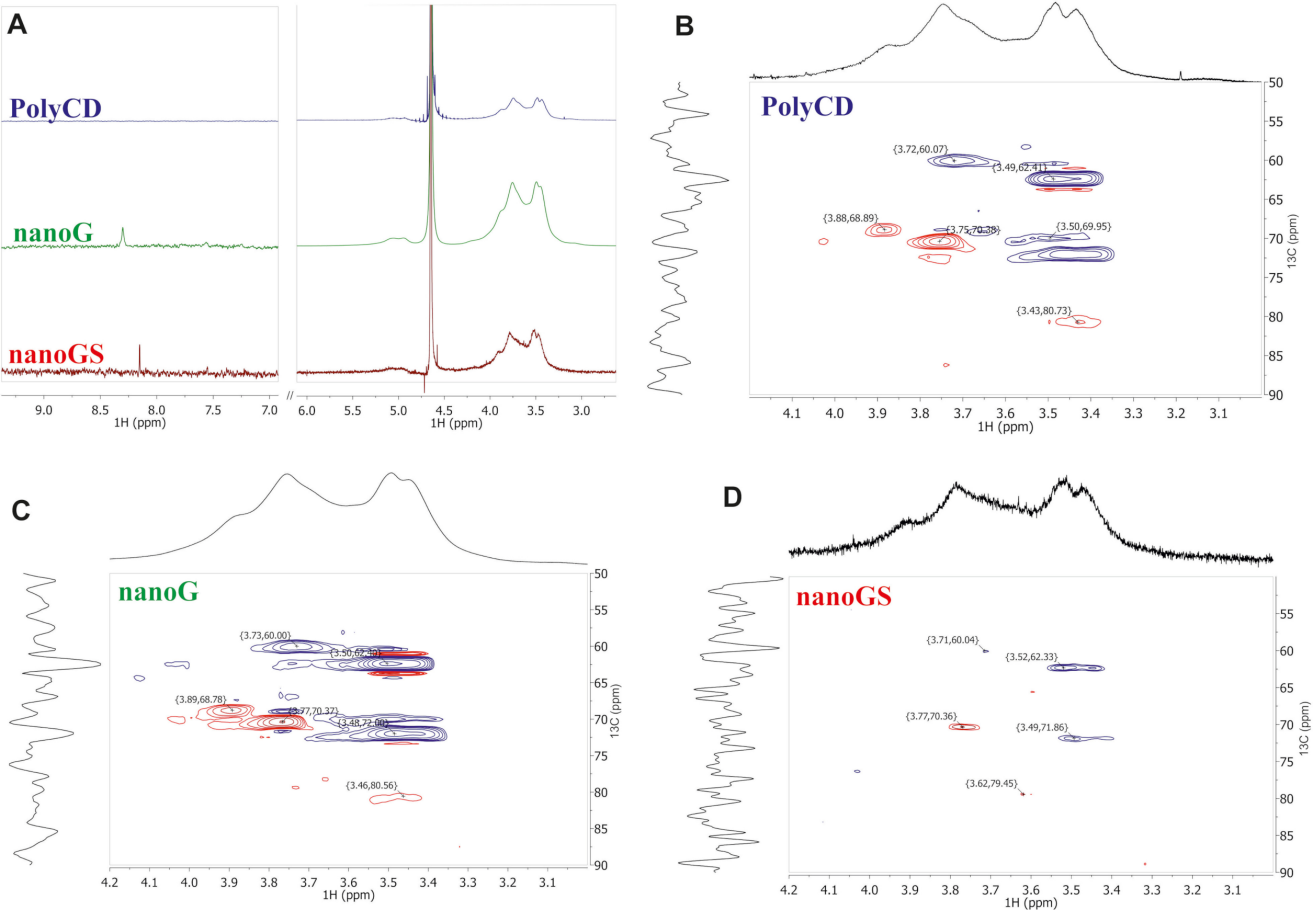
(A) ^1^H-NMR spectra of PolyCD, nanoG, and nanoGS. (B–D) Enlargement of DEPT-edited HSQC spectra of PolyCD, nanoG, and nanoGS, respectively (D_2_O, 298 K, 500 MHz).

To further investigate the extraordinary ability of PolyCD to produce and to stabilize gold-based NPs and to shed light on the mechanism of Au(III) reduction by PolyCD, NMR analyses were carried out. The colloidal solutions of nanoG and nanoGS were lyophilized and redispersed in deuterium oxide to perform NMR analyses. The ^1^H NMR spectra of PolyCD, nanoG, and nanoGS provided only limited structural information due to the complex structure of PolyCD and/or its eventual oxidated derivatives. The pattern of resonances attributed to the β-CD-branched polymer was very similar for all the samples. Specifically, two distinct broad regions of signals in the range between 5.0–5.5 ppm and 3.0–4.5 ppm attributed to the CD anomeric protons and to all the remaining resonances of the polymer, respectively, were detected (Figure 3A). Interestingly, after the formation of metallic Au NPs, the new diagnostic resonance at 8.30 ppm was detected in ^1^H NMR spectra of nanoG (Figure 3A, green trace). According to the literature, this signal suggests the formation of formic acid as a decomposition product during the gold reduction process in the carbohydrate/Au(III) system [25].

To obtain information about the PolyCD structural motives involved in the formation/stabilization of the NPs, DEPT-edited HSQC NMR spectra were recorded (Figure 3B–D). The most informative changes evidenced in the range of 3.2–4.2 ppm (Table 1) suggested that, in the formation/stabilization of the NPs, CD ring units (i.e., CD-C4 and CD-CH_2_OH) and the units of epichlorohydrin chain proximal to the CD ring (Figure 3, Table 1) are entangled. No significative changes were detected for epichlorohydrin chain units distal from the CD ring (i.e., –(CH_2_CHOHCH_2_O)*_n_*–).

**Table 1:** Informative changes evidenced in the range of 3.2–4.2 ppm and 60.04–80.73 for ^1^H and ^13^C spectra, respectively, by the spectral matching of PolyCD, nanoG, and nanoGS.

H-C correlation^a^	PolyCD (δ_H_, δ_C_)	nanoG (δ_H_, δ_C_)	nanoGS (δ_H_, δ_C_)

CD-CH_2_OCH_2_*CH*OH-R	3.88, 68.89	3.89, 68.78	3.90, 68.93
CD-C3/C5	3.75, 70.38	3.77, 70.37	3.77, 70.36
CD-*CH*_2_OH	3.72, 60.07	3.73, 60.00	3.71, 60.04
–(CH_2_CHOH*CH*_2_O)*_n_*–	3.49, 62.41	3.50, 62.40	3.55, 62.33
CD-CH_2_O*CH*_2_CHOH-R	3.50, 69.95	3.48, 72.00	3.49, 71.86
CD-C4	3.43, 80.73	3.46, 80.56	3.62, 79.45

^a^PolyCD H-C correlations were reported in [14].

**Figure 4:**
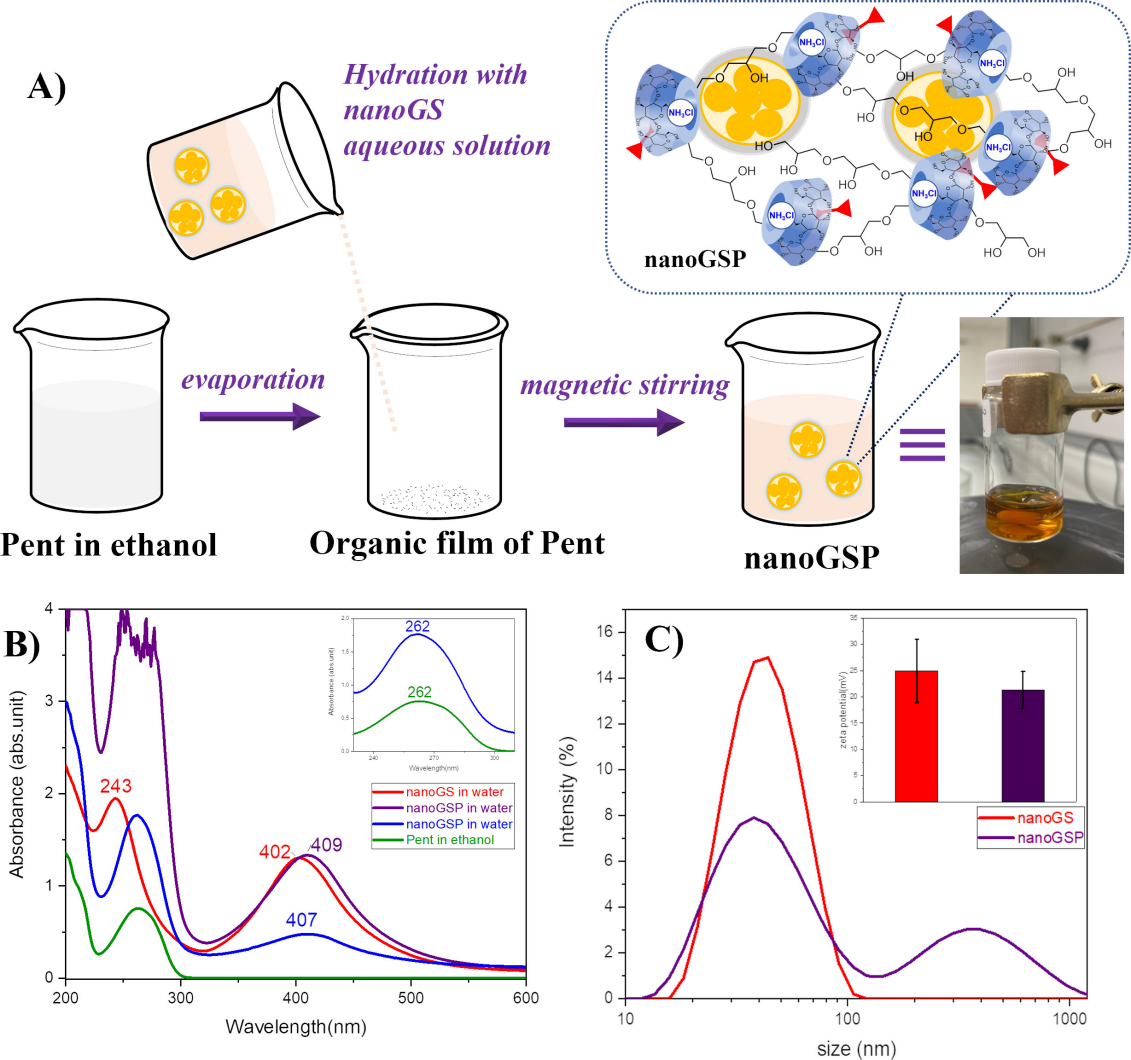
A) Schematic representation of nanoGSP preparation by supramolecular assembly. (B) UV–vis of nanoGS (red line), nanoGSP (violet line), diluted solution (4 times) of nanoGSP (blue line) in ultrapure aqueous solution, Pent solution in ethanol (green line, 52.5 µg/mL) *d* = 0.2 cm. (C) Size distribution (or *D*_H_) of nanoGS (red line) and nanoGSP (purple line) in a molar ratio of CD/Pent 1:1 in ultrapure water at 25 °C.

The supramolecular assembly between nanoGS and Pent was prepared at a 1:1 molar ratio using the classical method of organic film hydration (Figure 4A). In our study we employed Pent as a free base [19,26], slightly soluble in water (i.e., ≅30 µg/mL), instead of Pent isethionate salt used in the literature for the preparation of β-CD/Pent inclusion complex [27–28]. Specifically, De Paula et al. described a deep inclusion of Pent into CD cavities and demonstrated the interactions of both aromatic and aliphatic chain protons of Pent with internal hydrogens of the cavity by NMR analyses. As expected, weak interactions with the β-CD outer side were reported for the isethionate group [27]. Thus, in our experiments the use of Pent as a free base allowed us to verify its inclusion into the CD cavity excluding the unsought interactions of the isethionate group with the CD polymer and/or metal NPs.

The nanoGSP complex was characterized by UV–vis spectroscopy, DLS, and ζ potential measurements. The UV–vis spectra clearly indicated strong interactions between nanoGS and Pent (Figure 4B). Specifically, we can observe: i) the red shift in the LSPR band from 402 nm (nanoGS) to 409 nm (nanoGSP); (ii) the presence of the Pent band centred at 262 nm in nanoGSP; iii) a hyperchromic effect of the Pent band in nanoGSP, although it appears partially overlapped with the ascorbate band at 243 nm. The changes in the UV–vis spectra suggested the interactions of Pent with nanoGS components, and the increase of Pent absorbance in water clearly indicated the inclusion of the drug into CD cavities. Although we observed a red shift of the plasmonic band in UV–vis spectra (Figure 4B), the grafting of Pent to the silver surface was excluded by Raman analyses (Supporting Information File 1, Figure S1). Overall, these data suggested privileged interactions of Pent with CD cavities, whereas the Pent/Ag interactions can be excluded or, at least, they could be not strong enough to produce the Pent grafting.

The DLS and ζ-potential measurements indicated excellent stability of nanoGSP (Figure 4C and Table 2). Specifically, nanoGS and nanoGSP showed strong ζ-potential positive values (i.e., +25 and +21.3 mV, respectively). A single population of nanoscale objects with an average hydrodynamic diameter (*D*_H_) of 48 nm was detected for nanoGS, whereas two populations with a *D*_H_ of 47 nm (main population) and 415 nm (minor population) were observed for nanoGSP.

**Table 2:** DLS and ζ-potential measurements of nanoGS and nanoGSP.

Sample	*D*_H_ (nm ± SD)^a^ (%)^b^	ζ (mV ± SD)

nanoGS	44 ±16 (100%)	25.0 ± 6.0
nanoGSP	47 ± 25 (67.1%) 415 ± 210 (27.9%)	21.3 ± 3.5

^a^SD was calculated on three different batches. ^b^Mean size with corresponding intensity distribution (%). Micrometric aggregates (with an intensity distribution of about 5%) were also detected.

NanoGSP, together with nanoG, nanoGS, and Pent-free base as controls were tested against *L. infantum* according to previously reported procedures [26]. No significant antimicrobial effects were detected for nanoG, nanoGS, and NanoGSP, whereas an IC_50_ value of 5.66 µM against *L. infantum* was found for free Pent (Supporting Information File 1, Table S1), in line with previously reported data [19,21]. Cytotoxicity assays were also performed to assess the selectivity of the nanosystems (see hereinafter).

The inactivity of nanoG and nanoGS emerged in our work suggested that leishmanicidal effects described in the literature for MNPs or BMNPs [2,22–23,29] could be ascribed to their synergistic interactions with the phenolic components of natural extracts used as capping agents. Moreover, we supposed that, in nanoGSP, strong interactions between nanoGS and Pent, assisted by the CD cavities, prevented the release of Pent within 24 h. This is the time of the in vitro biological test and the activity of Pent included into the CD cavity should be better evaluated in vivo according to the literature [27]. To clarify this point, release experiments of PolyCD@Pent have been carried out in PBS (pH 7.4) at 37 °C using the dialysis method (Supporting Information File 1). The PolyCD@Pent complex (Supporting Information File 1, Figure S2) was prepared at [Pent]:[PolyCD] 1:1 molar ratio ([Pent] = [PolyCD] = 1 mM). Based on our experimental results, it is possible to estimate that the Pent released from PolyCD@Pent within 24 h is approx. 25% (Figure S3 in Supporting Information File 1).

## Conclusion

To sum up, we synthesized and fully characterized by spectroscopic and microscopic techniques bimetallic Au@Ag NPs with a core–shell architecture containing PolyCD as the capping agent. The combination of the antileishmanial drug pentamidine with Au@Ag NPs produced the nanosystem nanoGSP endowed with excellent stability as evidenced by UV–vis, DLS, and ζ-potential analyses. The role of PolyCD in the reduction/stabilization of NPs processes was investigated by NMR spectroscopy. The analyses of DEPT-edited HSQC NMR spectra evidenced the prominent role of CD ring units (CD-C4 and CD-CH_2_OH) and of the units of epichlorohydrin chain proximal to CD ring in the formation/stabilization of NPs. Moreover, for the first time, formic acid was identified as a by-product in the redox reaction of PolyCD/Au(III) system.

The inactivity of both mono- and bimetallic NPs (nanoG and nanoGS) against *L. infantum* parasites in our in vitro test suggested the crucial role of the capping agent for antileishmanial activities of NPs. Probably, in our case, the generation of reactive species described in literature for antileishmanial NPs was not synergically promoted by PolyCD. Moreover, we assumed that the biological inactivity of pentamidine in nanoGSP could be attributed to its not prompt availability due to strong interactions of the drug with CD cavities.

## Experimental

### General remarks

Tetrachloroauric acid (HAuCl_4_), silver nitrate (AgNO_3_), ascorbic acid, and pentamidine isethionate were commercially available (Merck). All reagents used (Merck) were used without further purification.

### Characterization techniques

UV–vis spectra were obtained on an Agilent model 8453 diode array spectrophotometer using 1 cm path length quartz cells (nanoG) and 0.2 cm path length quartz cells (nanoGS and nanoGSP) at room temperature (≈25 °C). The zeta potential, average hydrodynamic diameter, and width of distribution (polydispersity index, PDI) measurements were carried out by a Zetasizer Nano ZS (Malvern Instrument, Malvern, U.K.) at 25 °C in ultrapure water. The results are reported as the mean of three separate measurements ± the standard deviation (SD). The morphological characterization was performed using a high-resolution TecnaiG2 F20 XTWIN TEM with a 200 kV accelerating voltage. NMR spectra were recorded on a Varian 500 MHz spectrometer at rt ≈ 25 °C. The chemical shifts are expressed in ppm using acetone as an internal standard. NMR analyses and Raman analysis (Supporting Information File 1, Figure S1) were carried out according to previously reported protocols [14,30].

### Preparation of PolyCD Au NPs and PolyCD Au@Ag BMNPs

NanoG and nanoGS were prepared according to previously reported procedure [14]. Briefly, an aqueous solution of nanoG was prepared by mixing 1.5 mL of 10^−3^ M HAuCl_4_ and 9 mL of PolyCD aqueous solution (2 mg/mL water for HPLC) previously sonicated for 2 min and leaving the solution under stirring for 3 h, keeping the temperature in the range of 70–80 °C leading to the reduction of gold ions, visible by a pink colored solution. Before use, the PolyCD was purified by dialysis (membrane cutoff of 3.5 kDa) and lyophilized. The LSPR Au NPs peak at λ_max_ = 531 nm is detected in the UV–vis spectra of the pink solution (PolyCD = 1.71 mg/mL, Au = 28.17 µg/mL). PolyCD Au NPs, referred to as nanoGS, were stabilized for 12 h at room temperature before subsequent use. The synthesized nanoG was used as a seed material for subsequent silver shell growth, as follows: 40.5 μL of 10^−1^ M ascorbic acid and 13.5 µL of 10^−1^ M AgNO_3_ were added three times at room temperature, every hour, to a solution of nanoG (6.75 mL) to obtain nanoGS determined by a light orange staining of the solution. After each addition of silver ions, the mixture was kept under magnetic stirring at room temperature (20–25 °C) for 1 h. The reduction of silver ions was monitored by following the increase of the LSPR characteristic band at 402 nm. Before the NMR analyses, nanoG and nanoGS colloidal solutions were lyophilized and redispersed in D_2_O.

### Preparation of nanoGSP supramolecular assembly

NanoGSP was prepared by using the method of hydration of an organic film of pentamidine (Pent) at [PolyCD]:[Pent] 1:1 molar ratio. The organic film was obtained by slow evaporation of 1.36 mL of Pent ethanolic solution (1 mg/mL). The film was hydrated with nanoGS solution (4 mL) previously heated to 40 °C and the reaction mixture was left stirring at room temperature for 15 h.

### In vitro antileishmanial activity and cytotoxicity

For the antileishmanial activity, nanoG, nanoGS, and nanoGSP (all of them in aqueous solution) were evaluated in vitro against *L. infantum* MHOM/MA (BE)/67 intracellular amastigotes. The aqueous sample solutions (nanoG = 28.17 μg/mL of Au content; nanoGS = 27.51 μg/mL of Au content and 63 μg/mL of Ag content; nanoGSP = 27.51 μg/mL of Au content, 63 μg/mL of Ag content and 350 μg/mL of Pent content) were 10 × 2-fold diluted and these concentrations were then 20-fold diluted in the screening system. The highest in-test of Pent for nanoGSP was 17.5 μg/mL (or 51.4 μM). The Pent-free base (powder) was dissolved in 100% DMSO at 20 mM (stock solution). Then it was serially diluted in DMSO followed by a further (intermediate) dilution in demineralized water to ensure a final in-test DMSO concentration of <1%. The highest in-test concentration of Pent was 32 μM. Miltefosine was used as a reference drug. Cytotoxicity assays were performed both on primary peritoneal mouse macrophages (PMM) and human fetal lung fibroblasts (MCR-5) according to procedures previously described to assess selectivity [31]. Tamoxifen was employed as the reference drug. All obtained data are reported in the Supporting Information File 1.

## Supporting Information

File 1 Additional experimental data.
