# Supplementary material for: Supramolecular assembly of pentamidine and polymeric cyclodextrin bimetallic core–shell nanoarchitectures
Source: Beilstein J Nanotechnol. 2022 Nov 18;13:1361–9. doi: 10.3762/bjnano.13.112 (PMC9679597; doi:10.3762/bjnano.13.112)
Supplement: File 1 — Additional experimental data. [file Beilstein_J_Nanotechnol-13-1361-s001.pdf]

BEILSTEIN JOURNAL OF NANOTECHNOLOGY

Supporting Information

for

Supramolecular assembly of pentamidine and polymeric cyclodextrin bimetallic core–shell nanoarchitectures

Alexandru-Milentie Hada, Nina Burduja, Marco Abbate, Claudio Stagno, Guy Caljon, Louis Maes, Nicola Micale, Massimiliano Cordaro, Angela Scala, Antonino Mazzaglia and Anna Piperno

Beilstein J. Nanotechnol. **2022,** *13,* 1361–1369. doi:10.3762/bjnano.13.112

Additional experimental data

License and Terms: This is a supporting information file under the terms of the Creative Commons Attribution License (https://creativecommons.org/licenses/by/4.0). Please note that the reuse, redistribution and reproduction in particular requires that the author(s) and source are credited and that individual graphics may be subject to special legal provisions.
The license is subject to the *Beilstein Journal of Nanotechnology* terms and conditions: (https://www.beilstein-journals.org/bjnano/terms)

Materials and Methods

All the dispersions used for preparation and spectroscopic characterization of nanoassemblies were prepared in ultrapure water (Fresenius Kabi Italia) or in 10 mM phosphate buffer containing NaCl (137 mM) and KCl (2.7 mM) at pH 7.4 (PBS) at room temperature (rt ≈ 25 °C).

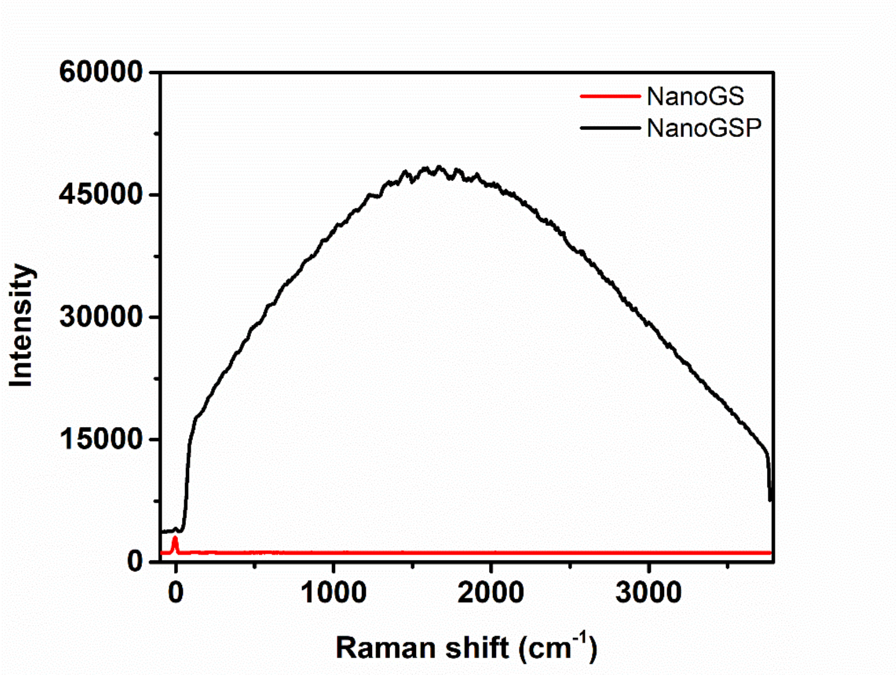

Figure S1: The Raman spectra of nanoGS and nanoGSP. The Raman spectra were recorded under an excitation of 532 nm. No specific Raman signal was detected.

Table S1: In vitro antileishmanial activity and cytotoxicity (IC_{50} μM and CC_{50} μM, respectively) of gold nanoparticles decorated with polycyclodextrins (nanoG), bimetallic Au-Ag core–shell nanoparticles (nanoGS), and bimetallic Au–Ag core–shell nanoparticles containing pentamidine (nanoGSP). Values represent the mean of two independent tests.

**Compound**	*L. infantum*[a]	PMM[b]	MRC-5[c]
**nanoG**	> 64.00	> 64.00	> 64.00
**nanoGS**	> 64.00	> 64.00	> 64.00
**nanoGSP**	> 51.40	> 51.40	> 51.40
**Pent**[d]	5.66	32.00	24.87
**Miltefosine**	3.17	–	–
**Tamoxifen**	–	–	13.60

[a] *Leishmania infantum* MHOM/MA/67/ITMAP263.

[b] Primary peritoneal mouse macrophages.

[c] Human fetal lung fibroblast (MRC-5) cell line toxicity.

[d] Pentamidine

Preparation of the PolyCD@Pent complex

The complex was prepared at [Pent]:[PolyCD] 1:1 molar ratio ([Pent] = [PolyCD] = 1 mM). The PolyCD was dissolved in ultrapure water (1.71 mg/mL) and sonicated in an ultrasonic bath (5 min). A thin organic film of free low Pent (0.35 mg/mL) was prepared by slow evaporation of an ethanol solution (EtOH) and the latter was hydrated at room temperature, with PolyCD dispersion followed by sonication in an ultrasonic bath (30 minutes). The diluted dispersions (Pent = 52.5 µg/mL, [Pent] = [CD] = 154 µM) were characterized by UV/Vis. For comparison, Pent/PolyCD nanocomplexes at the same concentration were prepared in PBS and analyzed by UV/Vis.

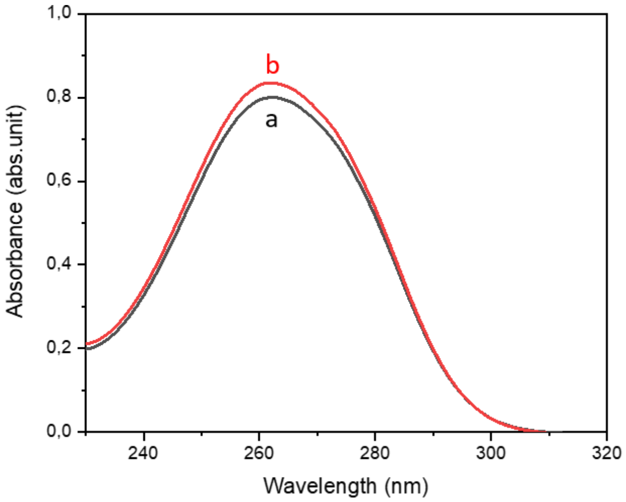

Figure S2: UV/Vis spectra of Pent (trace a) and of PolyCD@Pent in ultrapure water (trace b). Experimental conditions: [Pent] = 154 µM; d = 0.2 cm; T = 25 °C.

The Pent absorption profile shows a main band centered at 262 nm in ultrapure water which is also maintained in PolyCD@Pent even if in the latter there is an increase in absorbance at the same concentration. This was ascribed to an increased solubility of the drug upon interaction with PolyCD.

Release study of PolyCD@Pent

The Pent release profile from the PolyCD@Pent was evaluated in PBS at pH 7.4 by a dialysis method. PolyCD@Pent (Pent = 0.35 mg/mL), PolyCD concentrated solution

3.42 mg/mL (500 µL), and 500 µL of 10 mM PBS to a final volume of 1 mL were placed in a dialysis tube (spectra dialysis bags/Por®, MWCO 3500 kDa) and immersed in 5 mL of PBS (sink condition) under continuous stirring (250 rpm) at 37 ± 0.5 °C. At predetermined times, 1 mL of release medium was removed and replaced with an equal volume of fresh aqueous solution of PBS. The quantity of Pent released was evaluated by UV–vis spectroscopy (at λ = 262 nm) by using the extinction coefficient of Pent salt in PBS (ε ~ 26000 mol^{-1} cm^{-1}) and was expressed as a percentage ratio between the weight of Pent released and the total quantity of the drug.

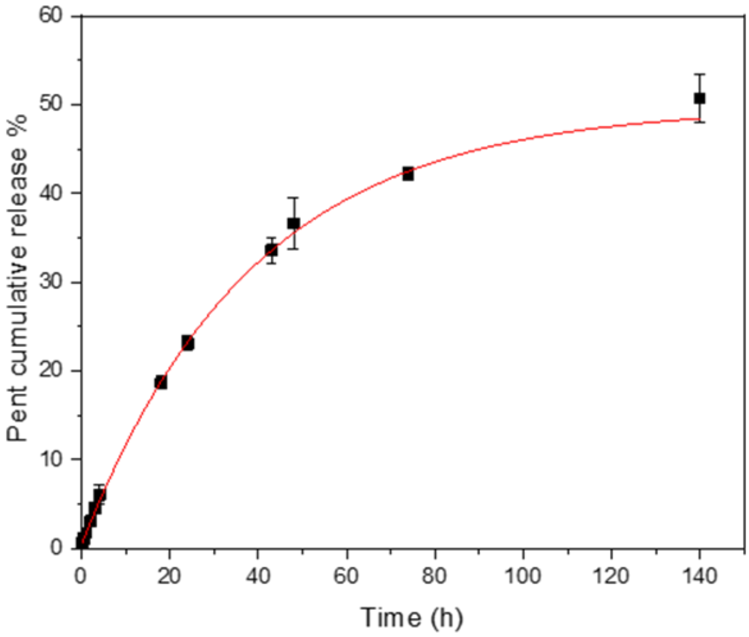

Figure S3: Release profile of Pent from PolyCD@Pent in PBS (pH 7.4, at 37 °C).

The amount of released Pent was determined in PBS by using UV–vis. No initial burst release was observed from PolyCD@Pent, with a slow and controlled release in time, leading to a final 53% of Pent in the external medium after 140 h (Figure S3).
